# Interaction effects of aging, word frequency, and predictability on saccade length in Chinese reading

**DOI:** 10.7717/peerj.8860

**Published:** 2020-04-01

**Authors:** Zhifang Liu, Wen Tong, Yongqiang Su

**Affiliations:** 1 Department of Psychology, Hangzhou Normal University, Hang Zhou, China; 2 Department of Psychology, Shanxi Normal University, Lin Fen, China; 3 Department of Psychology, Beijing Normal University, Bei Jing, China

**Keywords:** Chinese reading, Aging, Eye movements

## Abstract

**Background:**

It was well known that age has an impact on word processing (word frequency or predictability) in terms of fixating time during reading. However, little is known about whether or not age modulates these impacts on saccade behaviors in Chinese reading (i.e., length of incoming/outgoing saccades for a target word).

**Methods:**

Age groups, predictability, and frequency of target words were manipulated in the present study. A larger frequency effect on lexical accessing (i.e., gaze duration) and on context integration (i.e., go-past time, total reading time), as well as larger predictability effects on data of raw total reading time, were observed in older readers when compared with their young counterparts.

**Results:**

Effect of predictability and frequency on word skipping and re-fixating rate did not differ across the two age groups. Notably, reliable interaction effects of age, along with word predictability and/or frequency, on the length of the first incoming/outgoing saccade for a target word were also observed.

**Discussion:**

Our findings suggest that the word processing function of older Chinese readers in terms of saccade targeting declines with age.

## Introduction

It has been well documented that word processing may vary among young and older adult readers of Western languages such as English and German. Specifically, recent evidence has revealed a subtle decline in word identification among older adults when they read; that is, older adults spend more time fixating on target words, and regress to them more often than younger readers (Kemper, Crow & Kemtes, 2004; Laubrock, Kliegl & Engbert, 2006; Rayner, Castelhano & Yang, 2009; Rayner, Castelhano & Yang, 2010; McGowan et al., 2014; Whitford & Titone, 2017; Paterson, McGowan & Jordan, 2013a; Paterson, McGowan & Jordan, 2013b; Paterson, McGowan & Jordan, 2013c). Studies have also revealed that frequency and predictability effects, which are closely related to how easily any word can be processed, are also impacted by aging. For example, Kliegl et al. (2004) revealed that older readers yielded larger frequency effects, younger readers tended to skip more predictable words, and older adults re-fixated less on such words while reading in German. Rayner et al. (2006) also reported larger frequency effects for native older adult English readers than young adult readers; however, they failed to observe differences in predictability effects between the two age groups. Recently, Steen-Baker et al. (2017) reported a different contextual sensitivity in the regression patterns of older readers. A study conducted by Choi et al. (2017) revealed larger predictability effects on fixation time measures of target words based on age groups. Thus, it seemed that the eye movements of the two age groups might be affected by frequency/predictability factors in slightly different ways.

The visual and linguistic demands of written Chinese are very different from those of alphabetic texts. Even so, researchers have observed a similar difference in eye movement behaviors between young and older readers of Chinese; that is, studies have consistently revealed that older Chinese readers fixate on target words for a longer time than their younger counterparts (Zang et al., 2016). Wang and her collaborators replicated larger frequency and predictability effects on fixation time measures for older readers of Chinese when they read two-character-words than for their young counterparts (Wang et al., 2018a; Wang et al., 2018b; Zhao et al., 2019). However, there were still distinctions based on impact of age on eye movement control between readers of Chinese and alphabetic languages; this implies that older English adult readers demonstrate longer forward eye movements, and skip words more often than their younger counterparts (see, Rayner et al., 2006; Rayner, 2009), although, recent evidence has revealed that there is no difference between the two age groups in terms of skipping rate and saccade length (Choi et al., 2017). Growing evidence on the topic of Chinese reading has indicated that older readers make shorter forward saccades and skip words more infrequently than young adult readers (Wang et al., 2018a; Wang et al., 2018b; Zang et al., 2016; Li et al., 2018). Thus, it seems that older Chinese readers employ a more careful strategy for eye movements, as compared to older readers of alphabetic texts. Therefore, based on the findings of the aforementioned studies, it is explicit that the effect of age on eye movement behavior is both language specific and universal.

As an important aspect of eye movement control during reading, saccade targeting to word seems to differ across Chinese and alphabetic languages. For most alphabetic writing systems, readers project their eyes to a specific location of a word, which is called preferred-viewing location (PVL; Rayner, 1979). It has been confirmed that linguistic characteristics of a word, such as predictability, did not impact the initial landing position of the eyes for target words (Rayner et al., 2001). In contrast, previous studies failed to find reliable evidences that Chinese readers target saccade to PVL during reading. However, evidence has shown that a target word can easily be processed, in that their frequency and predictability modulate the lengths of saccades targeting. Specifically, Liu and his collaborators have confirmed that target-word frequency modulates the lengths of incoming and outgoing saccades for target words, with a shorter saccade to/from low-frequency word (Liu, Reichle & Li, 2015; Liu, Reichle & Li, 2016; Liu et al., 2017a; Liu et al., 2017b); when compared with less predictable target words, highly predictable target words elicited longer saccades (Liu et al., 2018). According to their findings, saccade amplitude is adjusted dynamically using lexical processing. Readers lengthen an impending saccade if the processing of the parafoveal or foveal word is easy (Liu, Reichle & Li, 2015; Liu et al., 2017a; Liu et al., 2017b).

It is known that older Chinese readers adopt a more careful saccade strategy during reading, which may be owing to their poorer word processing, possibly due to a visual decline with age and/or additional visual requirements for the Chinese text (Owsley, 2011; Zhang et al., 2012; Wang et al., 2018a; Wang et al., 2018b; Zang et al., 2016; Li et al., 2018). Previous studies have revealed that word length produced a similar landing position, probability, and duration of eye movement in both young and older adult readers of English (Rayner et al., 2006; Paterson et al., 2015). Von der Malsburg, Kliegl & Vasishth (2015) also observed an insignificant interaction effect of aging with word length on scan-path, whereas a Chinese study recently revealed a lager word length effect on older Chinese readers than their young counterparts both in terms of fixation time and saccade length (Li et al., 2018). As has been noted earlier, growing evidence on how age impacts Chinese reading has consistently shown that older readers yield larger frequency and predictability effects on fixation time (i.e., gaze duration and total reading time). However, little is known about whether or not this impact of age could be categorized into saccades behaviors (i.e., length of impeding incoming/outgoing saccades for target words). Do these two effects of linguistic characteristics on saccades length change with age in Chinese readers? Exploring this would be undeniably helpful for revealing the factors responsible for the effect of age on eye guidance in Chinese readers. In the present study, we focus on how age mediates the effects of word frequency and predictability on the lengths of impending incoming/outgoing saccades for a target word. How is the length of the impending saccade related to parafoveal and foveal processing (Liu, Reichle & Li, 2015; Liu et al., 2018)? Despite research conducted by Liu and his collaborators investigating the individual effects of word predictability and frequency on the length of impending saccades among a group of young adults (Liu, Reichle & Li, 2015; Liu, Reichle & Li, 2016; Liu et al., 2017a; Liu et al., 2017b), questions regarding how these two factors contribute, additionally or interactively, to saccade length, and whether or not age impacts these effects, still persist. Prior literature has suggested the need to predict differences in basic eye movement in terms of age; currently, it suggests that older adults need more time, fixate longer, and exhibit short saccade lengths during sentence comprehension as compared to young adults. However, we have mainly focused on the different effects of frequency/predictability or their interaction on the length of impending saccades for old adult readers. Encouraged by previous studies, we manipulated predictability and frequency orthogonally. The impact of age on lexical processing produced larger effects of frequency/predictability or their interactions on the fixation time of older readers. As the focus of present study, it predicates reliable interaction effects of age with two linguistic characteristics of words (i.e., frequency & predictability) and their interaction on the length of impending saccades.

## Methods

### Ethical consideration

The Center for Cognition and Brain Disorders of Hangzhou Normal University granted ethical approval to carry out the study within its facilities (Approval Number, 20190408). Data were anonymously collected after participants provided written informed consent by signing a form prior to their participation.

### Participants

Eighty young adults from Shanxi Normal University, and 40 older adults participated in the experiments. Older adults were recruited from the local community and included retired university teaching staff. All older adult participants were aged over 60 years ( *M* = 62.40, SD = 2.01). Young adults included students from Shanxi Normal University. These two groups did not differ in number of years of schooling (young adults: *M* = 13.38, SD = 0.49; old adults: *M* = 13.43, SD = 1.96, *t* =  − 0.216, *p* > 0.05). All participants were right-handed, had normal or corrected-to-normal vision; there was no difference between the two age groups in terms of corrected vision which was measured using the Tumbling E acuity chart (older: *M* = 4.98, SE = 0.121; younger: *M* = 4.99, SE = 0.08; *t* = 0.269, *p* > 0.05). All of them were native Chinese speakers. After completing the reading tasks, they were paid ¥30 for participation.

### Apparatus

An Eye Link II device, which was manufactured by SR Research Ltd., was used to record eye movements of the participants. It is a kind of infrared video-based tracking system. The camera of this device samples at a rate of 500 Hz. The sentence stimuli were presented in black against a white background. Participants sat 45 cm away from the monitors, which were 19-inch DELL LCD devices with a refresh rate of 60 Hz and 1024 ×768 pixel resolution. The sentences were displayed in Song font, with each Chinese character subtending approximately a 1.32 degree visual angle.

### Design and stimuli

The experiment followed a 2 (frequency of target words: high vs low) ×2 (predictability of target words: predictable vs unpredictable) ×2 (group: young adults vs old adults) design. Participants read 40 framed sentences which contained the target words. Example of these sentences are shown in Table 1. All the target words were composed of two characters, in which half of the these were high-frequency words, and another half were low-frequency words. Word and character frequencies were calculated using occurrences per million characters as a standardized measure, based on the database of Modern Chinese corpus word frequency and the database of Modern Chinese corpus character frequency, respectively, which were available at http://corpus.zhonghuayuwen.org/. The cut-off for high-frequency target words was more than 50 occurrences per million characters, while that for low-frequency words was less than 5 occurrences per million characters. As seen in Table 2, the mean frequency of words in the high-frequency condition is about 30 times that of the mean frequency in the low-frequency condition. Thus, the word frequency manipulations in the current study were wider than those in previous studies (Wang et al., 2018a; Wang et al., 2018b). In addition, half of the target words were predictable from prior context, while half were unpredictable from the prior context. A group of 19 participants who did not participate in the experiment were asked to assess the predictability of the target words. They were shown the sentence frame up to, but not including, the target word and asked to complete the sentence. It was found that predictable target words were generated 74% of the time, whereas unpredictable target words were generated less than 1% of the time.

**Table 1:** Example sentences.

Conditions	Sentence
HF–P	公司经理在提高产品质量方面花费了大量精力。 In older to improving product ***quality***, the company manager put a lot of effort
LF–U	公司经理在提高产品名声方面花费了大量精力。 In older to improving product ***fame***, the company manager put a lot of effort
LF–P	外星人经常驾驶飞船去往地球的各个角落。 Aliens often drive ***spacecraft*** to all corners of the earth.
HF–U	外星人经常驾驶汽车去往地球的各个角落。 Aliens often drive ***car*** to all corners of the earth.

**Notes.**

HF high-frequency targets P predictable targets U unpredictable targets LF low-frequency targets

**Table 2:** The characters of target words (predictable, frequency, and stroke).

Conditions	Word predictable	Word frequency	First character frequency	Second character frequency	First character strokes	Second character strokes
HF–P	0.74(0.14)	111.60(62.59)	792.27 (537.11)	597.95(435.01)	7.45(2.87)	7.60(2.68)
LF–U	0.01(0.01)	3.42(0.72)	798.27(1,038.02)	684.40(545.69)	7.25(3.54)	7.25(2.17)
LF–P	0.74(0.17)	3.69(1.30)	630.24(818.67)	554.74(571.21)	7.60(2.60)	7.65(2.52)
HF–U	0.01(0.02)	113.94(60.21)	729.21(531.07)	768.29(580.95)	7.20(2.53)	7.65(1.53)

**Notes.**

The standard deviations are given in parentheses.

All four word types were balanced in terms of character frequency and strokes (*ps* > 0.05), as seen in Table 2. There were no differences in word predictability, between predictable high frequency target words (HF-P) and predictable low frequency target words (LF-P), and between unpredictable low frequency target words (LF-U) and unpredictable high frequency target words (HF-U; *ps*>0.05), nor were there differences in word frequency between HF-P and HF-U, and between LF-U and LF-P. As seen in Tables 1 and 2, we developed two kinds of framed sentences. The first contained HF-P and LF-U target words, and the second contained LF-P and HF-U target words. It should be noted that words prior to the target word were also two-characters in length, and were also balanced in terms of word frequency, character frequency, and strokes. The characters of pretarget words are shown in Table 3. There was no difference between the two kinds of prior target words in terms of word/character frequency and strokes (*ps*>0.05). Fillers were not used in the present study.

**Table 3:** The frequency, and stroke characters of pretarget word.

Pretarget word in frame	Word frequency	First character frequency	Second character frequency	First character strokes	Second character strokes
HF-P LF-U	75.15(84.06)	818.81(671.05)	1,285.75(1,021.67)	7.40(1.85)	7.30(2.81)
LF-P HF-U	65.58(72.83)	649.24(698.92)	1,331.60(961.52)	7.90(1.89)	7.50(2.06)

**Notes.**

The standard deviations are given in parentheses.

### Procedure

When participants arrived, they were instructed to read sentences silently to understand their meaning and respond manually to comprehension questions. Then, experiment began with a 3-horizontal-point calibration followed by 12 practice trials and 40 experimental sentences. All 40 experimental sentences were sampled using a Latin square to ensure that these sentences were shown in equal frequency in the 4 conditions. These 40 experimental sentences were displayed in a randomized order. The sentences were replaced during 28.6% of trials after a comprehension question with a YES or NO response (a total of 16 comprehension questions, with equal numbers of YES and NO answers). The eye tracker was engaged for a new calibration prior to each trial to ensure accuracy of eye tracing data if necessary; that is, we would conduct a re-calibration if error from drift correction of the present trial was greater than 0.5°. The entire experiment was completed in less than half an hour.

### Data analysis

Comprehension accuracy was lower for older adults than their younger counterparts (94.5% vs % 77.3%, *p* < 0.001). This may be partly owing to older adults having difficulty with manual responses, even when they know the right answers (Wang et al., 2018b). We computed the correlations between older adults’ comprehension accuracy, with their reading time for sentences, and global measures of their eye movements (all *rs*<0.25, all *ps* > 0.05). Therefore, these findings indicated that the variations of older readers’ comprehension scores did not produce qualitative differences in reading performance or eye movement behavior, and there was no evidence that indicated differences in reading strategy between high- and low-scoring older readers. Eye movements at sentence-level and word-level were analyzed using the R Statistics Package. Continuous data were analyzed using the LMM, and binary variables were analyzed using GLMM. Complicated models including random slopes posed a problem of convergence; therefore, we used maximal random effect structures, as suggested by Barr et al. (2013), with participants and stimuli as crossed random effects. Age group was the only fixed factor for the sentence-level measures analysis. Age group, frequency, predictability, and their interactions were treated as fixed factors for word-level measures analysis (coded as sum contrasts -1/2 vs 1/2 for young and old adults, predictable vs unpredictable, and for high and low frequency). Contrasts in the main effects were defined using sliding contrasts in the MASS package (Venables & Ripley, 2002). Effects based on both raw and log10-transformation were reported for continuous variables. Regression coefficients (*b*), standard errors (*SE*), *t* (*t* = *b/SE*), and *p* values were reported. Models were fitted with the lme4 package (ver. 1.1-19; (Bates et al., 2015) and *p*-values were estimated with the lmerTest package (ver. 3.0-1) in R (ver. 3.5.2; R Development Core Team, 2016). When analyzing raw data of gaze duration and log-transformed data of total reading time, no convergence was observed in the maximal random effect structures model, and so the models used for analyzing these data only included random effect of participants.

Eye movements at sentence-level and word-level were recorded. Sentence-level measures included sentence reading time (SRT), average fixation duration (AFD; mean duration of all fixation while reading a sentence), fixation count (FC), average saccade length (ASA; mean length of all saccades), regression number (RegNO; backward saccades number), word-level measures comprising fixation (time/probability), and saccade measure (length) of target words. Fixation time measures for target words included first fixation duration (FFD; the duration of the first fixation on the word irrespective of the number of fixations), gaze duration (GD; the sum of all fixations’ duration on the word before moving to another word), and total reading time (TRT; sum of all fixations’ duration). Go-past time (Go-past) is the sum of fixation duration from when the current area of interest is first fixated upon until one’s eyes enter an interest area with a higher id. FFD and GD were treated as measures of first-pass reading, and TRT was treated as a measure of second-pass reading because it includes regressive re-reading time. Go-past was treated as a measure of difficulty in integrating a target word with the context before moving on in a sentence. Fixation probability measures were associated with target words, and included the probability of skipping (Skip.pro) or re-fixating (Refix.pro) on a target word. Saccade measures comprised incoming saccade length (ISL; length of the first-pass progressive saccade resulting in fixation on the target word) and outgoing saccades length (OSL; length of the first-pass progressive saccade launched away from the target word).

## Results

### Sentence-level measures analyses

The mean and standard errors of the sentence level measures are shown in Table 4, and corresponding statistical effects are summarized in Table 5. The results show that an older adult requires longer fixations and regressions to comprehend a sentence than their young counterparts. The pattern of these effects is consistent with the fact that older adults experience greater difficulty in reading (Wang et al., 2018a; Wang et al., 2018b; Zang et al., 2016). However, contrary to what we had expected, we did not observe any significant differences in the average saccade length between the two age groups. As previous studies on effects of age on Chinese readers have also shown no differences in age groups in terms of saccade length, the our findings regarding sentence-level measures were consistent with previous research (Zhao et al., 2019; Li et al., 2018).

**Table 4:** The mean and standard errors of the sentence level measures across age groups.

	SRT	AFD	FC	ASA	RegNo
Young	3,558(165.6)	226(3.3)	13.5(0.56)	2.16(0.06)	3.6(0.18)
Older	5541(234.1)	247(4.7)	20.0(0.79)	2.11(0.08)	4.8(0.25)

**Notes.**

Means and standard errors are computed across subjects means. The standard errors are given in parentheses.

SRT sentence reading time in millisecond AFD mean fixation duration in milliseconds FC fixation number ASA mean saccade length in character RegNO number of regressions

**Table 5:** Linear mixed-effects model analyses on sentence level measures.

	SRT				SRT log-transformed			
	*b*	*SE*	*t*	*p*	*b*	*SE*	*t*	*p*
Inter	4,551.5	163.7	27.81	<0.001	3.601	0.017	214.695	<0.001
Group	−1987.1	286.7	−6.93	<0.001	−0.201	0.030	−6.785	<0.001
	AFD				AFD log-transformed			
Inter	236.296	2.916	81.041	<0.001	2.367	0.005	454.054	<0.001
Group	−20.778	5.704	−3.642	<0.001	−0.041	0.010	−4.027	<0.001
	ASA				ASA log-transformed			
Inter	2.815	0.074	38.166	<0.001	0.426	0.011	37.76	<0.001
Group	0.062	0.136	0.454	0.65	0.020	0.021	0.97	0.334
	FC				FC log-transformed			
Inter	16.728	0.554	30.208	<0.001	1.176	0.015	78.361	<0.001
Group	−6.497	0.963	−6.745	<0.001	−0.169	0.026	−6.463	<0.001
	RegNO				RegNO log-transformed			
Inter	4.168	0.178	23.47	<0.001	0.537	0.018	29.431	<0.001
Group	−1.200	0.31	−3.87	<0.001	−0.104	0.032	−3.223	0.002

### Word-level measures analyses

#### Fixation time

A total of 4,268 observations contributed to the analyses. As shown in Tables 6 and 7, differences were observed between the two age groups of readers in terms of fixation time measures. Specifically, older readers fixated on target words longer than young readers. Reliable effects of predictability were observed; specifically, predictable targets were fixated upon for a shorter time than unpredictable targets. A similar pattern was observed in the effect of frequency also, wherein frequency had a significant impact on FFD, GD, Go-past, and TRT. The present observations on the interaction effects are also important in the present study. Reliable interaction effect of Predictability × Frequency on raw GD was due to the larger frequency effect of unpredictability as compared to predictability (39 ms vs 22 ms). Reliable interaction effects of Group × Predictability and Group × Frequency on raw TRT were due to consistently large frequency and predictability effects on older adult readers than on young adult readers (group differences of frequency effect: 33 ms *vs* 75 ms; group differences of predictability effect: 117 ms *vs* 163 ms). Reliable interaction effects of Group × Frequency on both raw and log-transformed GD as well as go-past were due to the larger frequency effects on older readers as compared to younger readers (frequency effects for older: GD = 53 ms; go-past = 76 ms; frequency effects for young adult readers: GD = 17 ms; go-past = 17 ms)

**Table 6:** Means and standard errors of eye movement measures on target words across conditions and age groups.

Measures	Young adults readers				Older adults readers			
	HF–P	LF–P	HF–U	LF–U	HF–P	LF–P	HF–U	LF–U
FFD	232(5.2)	234(5.2)	243(5.2)	252(5.2)	256(7.4)	266(7.4)	266(7.4)	280(7.4)
DG	254(8.7)	261(8.7)	275(8.7)	299(8.7)	302(12.3)	342(12.3)	336(12.3)	399(12.3)
Go-Past	349(15.9)	364(15.9)	447(15.9)	458(15.9)	365(22.5)	454(22.5)	467(22.5)	527(22.5)
TRT	287(18.8)	314(18.8)	399(18.8)	437(18.8)	367(26.6)	447(26.6)	534(26.6)	605(26.6)
Skip.Pro	24.9(1.8)	24.4(1.8)	21.1(1.8)	20.8(1.8)	11.8(2.6)	12.0(2.6)	12.8(2.6)	8.7(2.6)
Refix.pro	8.9(1.8)	10.5(1.8)	11.4(1.8)	16.6(1.8)	17.8(2.6)	27.0(2.6)	23.0(2.6)	31.3(2.6)
ISL	2.00(0.10)	2.04(0.10)	1.99(0.10)	1.91(0.10)	1.81(0.14)	1.75(0.14)	2.02(0.14)	1.89(0.14)
OSL	1.87(0.07)	1.81(0.07)	1.89(0.07)	1.80(0.07)	1.81(0.09)	1.79(0.09)	1.64(0.09)	1.85(0.09)

**Notes.**

Means and standard errors are computed across subjects means, standard errors are shown in parentheses, time measures are in milliseconds, probability measures are in %, saccade length are in character spaces.

**Table 7:** Linear mixed-effects model analyses on fixation time measures.

	FFD				FFD log-transformed			
	*b*	*SE*	*t*	*p*	*b*	*SE*	*t*	*p*
Inter	253.356	4.109	61.660	<0.001	2.372	0.007	338.994	<0.001
F	−8.917	2.896	−3.079	0.002	−0.015	0.005	−2.97	0.003
P	−13.618	2.896	−4.702	<0.001	−0.024	0.005	−4.634	<0.001
G	−27.459	7.397	3.712	<0.001	−0.052	0.012	−4.221	<0.001
F × P	4.310	9.210	0.468	0.642	0.008	0.017	0.473	0.638
F × G	6.421	5.791	1.109	0.268	0.006	0.010	0.610	0.542
P × G	−1.119	5.793	−0.193	0.847	−0.005	0.010	−0.454	0.65
P × F × G	0.419	11.585	0.036	0.971	0.004	0.021	0.209	0.834
	GD				GD log-transformed			
	*b*	*SE*	*t*	*p*	*b*	*SE*	*t*	*p*
Inter	308.821	5.863	52.676	<0.001	2.435	0.010	255.162	<0.001
F	−35.235	4.913	−7.171	<0.001	−0.037	0.006	−6.115	<0.001
P	−38.56	4.914	−7.846	<0.001	−0.045	0.006	−7.363	<0.001
G	−72.741	11.725	−6.204	<0.001	−0.098	0.015	−6.504	<0.001
F × P	19.22	9.827	1.956	0.051	0.019	0.026	0.732	0.469
F × G	36.577	9.827	3.722	<0.001	0.027	0.012	2.164	0.031
P × G	16.512	9.829	1.680	0.093	0.009	0.012	0.77	0.442
P × F × G	−7.645	19.654	−0.389	0.697	0.012	0.024	0.494	0.621
	Go-past				Go-past log-transformed			
	*b*	*SE*	*t*	*p*	*b*	*SE*	*t*	*p*
Inter	428.841	15.106	28.389	<0.001	2.542	0.012	205.264	<0.001
F	−45.922	9.844	−4.665	<0.001	−0.042	0.008	−5.305	<0.001
P	−93.263	9.847	−9.472	<0.001	−0.081	0.008	−10.171	<0.001
G	−50.643	21.167	−2.393	0.018	−0.055	0.018	−3.122	0.002
F × P	−18.555	47.399	−0.391	0.698	−0.015	0.038	−0.398	0.693
F × G	60.976	19.690	3.097	0.002	0.055	0.016	3.431	<0.001
P × G	−5.006	19.694	−0.254	0.799	−0.007	0.016	−0.447	0.655
P × F × G	19.619	39.386	0.498	0.618	−0.002	0.032	−0.002	0.941
	TRT				TRT log-transformed			
	*b*	*SE*	*t*	*p*	*b*	*SE*	*t*	*p*
Inter	423.663	18.093	23.415	<0.001	2.574	0.010	246.338	<0.001
F	−53.903	9.032	−5.968	<0.001	−0.043	0.008	−5.510	<0.001
P	−140.059	9.032	−15.507	<0.001	−0.114	0.008	−14.551	<0.001
G	−128.83	27.043	−4.764	<0.001	0.097	0.021	−4.648	<0.001
F × P	0.326	51.371	0.006	0.995	−0.011	0.016	−0.724	0.469
F × G	42.285	18.065	2.341	0.019	0.025	0.016	1.579	0.114
P × G	45.491	18.065	2.518	0.012	0.017	0.016	1.117	0.264
P × F × G	19.5525	36.129	0.541	0.588	0.017	0.031	0.539	0.59

**Notes.**

F frequency factors P predictable factors G group factors

#### Probability measures

A total of 4,800 observations contributed to the analyses. As shown in Tables 6 and 8, the two age groups of readers significantly differed in the probability of skipping and re-fixating on target words. Older readers skipped target words less often, and re-fixated on them more than younger readers. Significant predictability effects were found on Skip.Pro and Refix.Pro, with a higher skip and less re-fixation probability for predictable as compared to unpredictable words. Frequency effect was reliable only on Refix.pro. No interaction effects were reliable on any probability measures.

**Table 8:** Linear mixed-effects model analyses on probability and saccade measures.

	Skipping				Re-fixating			
	*b*	*SE*	*Z*	*p*	*b*	*SE*	*Z*	*p*
Inter	−1.953	0.112	−17.372	<0.001	−1.945	0.135	−14.4	<0.001
F	0.128	0.094	1.354	0.176	−0.459	0.087	−5.275	<0.001
P	0.192	0.094	2.041	0.041	−0.373	0.087	−4.294	<0.001
G	1.137	0.206	5.524	<0.001	−0.948	0.222	−4.262	<0.001
P × F	−0.253	0.247	−1.025	0.305	0.086	0.339	0.254	0.799
F × G	−0.200	0.188	−1.059	0.290	0.238	0.174	1.373	0.17
P × G	0.080	0.188	0.426	0.67	−0.125	0.173	−0.723	0.47
P × F × G	0.512	0.377	1.359	0.174	0.426	0.347	1.229	0.219
	ISL				ISL log-transformed			
	*b*	*SE*	*t*	*p*	*b*	*SE*	*t*	*p*
Inter	1.922	0.078	24.618	<0.001	0.214	0.015	14.392	<0.001
F	−0.059	0.037	−1.597	0.110	0.007	0.007	1.066	0.287
P	0.031	0.037	−0.837	0.403	−0.0003	0.007	−0.047	0.962
G	−0.121	0.150	−0.830	0.408	0.016	0.027	0.596	0.553
P × F	−0.081	0.133	−0.611	0.545	−0.022	0.030	−0.747	0.459
F × G	−0.038	0.073	−0.524	0.601	0.002	0.014	0.165	0.869
P × G	0.219	0.073	2.992	0.003	0.030	0.014	2.173	0.030
P × F × G	0.061	0.147	0.414	0.679	−0.065	0.027	−2.370	0.018
	OSL				OSL log-transformed			
	*b*	*SE*	*t*	*p*	*b*	*SE*	*t*	*p*
Inter	1.793	0.057	31.696	<0.001	0.209	0.012	17.059	<0.001
F	0.011	0.034	0.316	0.752	0.014	0.006	2.441	0.015
P	0.04	0.034	1.190	0.234	0.020	0.006	3.603	<0.001
G	0.079	0.095	0.833	0.407	0.025	0.028	1.177	0.242
P × F	0.101	0.141	0.716	0.478	0.020	0.028	0.723	0.474
F × G	0.148	0.067	2.201	0.028	0.023	0.011	2.102	0.036
P × G	−0.081	0.067	−1.205	0.228	−0.014	0.011	−1.295	0.195
P × F × G	0.264	0.134	1.956	0.051	−0.063	0.022	−2.82	0.005

**Notes.**

F frequency factors P predictable factors G group factors

#### Saccade length

A total of 3,908 and 3,441 observations contributed to ISL and OSL analyses, respectively. As shown in Tables 6 and 8, analyses revealed no significant effects of group, frequency, and predictability on raw data of both ISL and OSL, but reliable frequency effects on log-transformed OSL was observed. Reliable interaction effects of Group × Predictability on ISL was due to a negative predictability effect on young adult readers (−0.08 char), but a reliable positive effect on older readers (0.14 char). As shown in Table 8, a three-way interaction (Group × Predictability × Frequency) in log-transformed ISL was observed. We employed models for analyzing the data of log-transformed ISL for younger and older readers separately to clarify this interaction, and observed opposite values for these two groups, although both interactions of Predictability × Frequency were not reliable (young readers: *b* =  − 0.054, *SE* = 0.039, *t* =  − 1.391, *p* = 0.172; maximal random effect structures model did not converge for older readers, so this model on only included the random effect of participants: *b* = 0.013, *SE* = 0.017, *t* = 0.748, *p* = 0.454). As for OSL, reliable interaction effects of Group × Frequency was also due to a negative effect of frequency on the young readers (-0.06 char) and a positive effect on older readers (0.08 char). To further investigate the three-way interaction (Group × Predictability × Frequency) in OSL, we employed models for analyzing the data of younger and older readers separately, which also yielded opposite interaction values of Predictability × Frequency for these two groups (results of raw OSL for young readers: *b* =  − 0.026, *SE* = 0.157, *t* =  − 0.168, *p* = 0.867; results of raw OSL for older readers: *b* = 0.234, *SE* = 0.185, *t* = 1.265, *p* = 0.213; results of log-transformed OSL for young readers: *b* =  − 0.010, *SE* = 0.035, *t* =  − 0.287, *p* = 0.776; results of log-transformed OSL for older readers: *b* = 0.052, *SE* = 0.024, *t* = 2.168, *p* = 0.036), all of these interactions were not reliable.

## Discussion

The present study assessed how the effects of frequency and predictability, and their interaction in eye movement behavior, especially in case of saccades, on Chinese readers were mediated by age. This study contributes to the growing evidence on the impact of age on word processing in several ways. First, the results of the word-level measures analyses replicated those of previous studies, which state that older readers need more fixation time for comprehending text. However, we could not determine the effect of age on average saccade length, which is also consistent with the scope of the findings of a recent study by Zhao et al. (2019). Second, with regards to the fixation time measures on target words, older readers fixated on these words for a longer time and re-fixated on them more often than younger readers, which also mirrors previous findings of studies on both Chinese and alphabetic text reading (Kliegl et al., 2004; Rayner et al., 2006; Rayner, 2009; Rayner, Castelhano & Yang, 2010; Kemper & McDowd, 2006; Kemper & Liu, 2007; Paterson, McGowan & Jordan, 2013a; Paterson, McGowan & Jordan, 2013b; Paterson, McGowan & Jordan, 2013c; McGowan et al., 2014; Zang et al., 2016; Wang et al., 2018a; Wang et al., 2018b). Third, we replicated age-related differences in skip measure; specifically, older Chinese readers skipped target words less often than young adult readers (Zang et al., 2016; Wang et al., 2018a; Wang et al., 2018b). Moreover, we observed the reliable impact of age on frequency and predictability, and their interaction effects on certain eye movement measures, which notably reveal eye movement control and word processing in Chinese reading.

For the interaction effects involving age with the linguistic characteristics of predictability and frequency, our findings further suggest that age promotes the effects of frequency. Previous studies have revealed larger frequency effects on fixation time measures, such as first fixation duration, gaze duration, and total reading time, in alphabetic text reading (Kliegl et al., 2004; Rayner et al., 2006). Conversely, Wang and her collaborators failed to report any larger frequency effects on older Chinese readers when analyzing log-transformed fixation time; however, they reported larger frequency effect on raw fixation time measures, such as gaze duration and total reading time, which was owing to older readers demonstrating very long fixation durations for low frequency words (Wang et al., 2018a; Wang et al., 2018b). The interaction effect between age group and frequency is extremely difficult to examine. Analyses on log-transformed data are likely to distort the ratio scale properties, which can lead to effects impacted by distribution tail to go unnoticed (Wagenmakers et al., 2012; Lo & Andrew, 2015); therefore, as compared to log-transformed fixation data, analyzing raw data is more convenient for detecting such interactions. However, we observed a larger frequency effect on log-transformed gaze duration and go-past time for older Chinese readers, which may partially be owing to a much more potent manipulation of word frequency.

Larger predictability effects on older Chinese readers were observed in fixation time measures, which suggest that older readers make greater use of context information for word identification than their young counterparts (Zhao et al., 2019). Our findings also help understand the role of age in utilizing context cues to promote eye movement and word processing during reading. We could not determine a reliable interaction effect of age and predictability on fixation measures in the first fixation duration and go-past time, but marginal or reliable interaction effects on raw data of gaze duration and total reading were observed. Interaction effects involving older groups with other variables are not easy to examine, but raw data help in detecting such interactions. Therefore, our findings have contributed, at least partly, to the evidence that older readers make greater use of context to identify words during reading (Choi et al., 2017; Zhao et al., 2019). Our results, of course, also further elaborate on how predictability impacts word identification in its interaction with frequency. Despite many observations by studies on eye tracking regarding the contributions of predictability and frequency to fixation durations while reading alphabetic text (Kennedy et al., 2013; Rayner et al., 2004), questions regarding whether these two factors contribute additionally, or interactively, to eye movement control in Chinese reading, and whether age impacts their interactions, still persist. Our results demonstrate the additional contributions of predictability and frequency on all measures of fixation for both age groups. Thus, our findings suggest that, at least for older readers, interaction effects of frequency and predictability on fixation time appears to be similar across scripts and age groups.

The probability of skipping and re-fixating on target words revealed some differences between Chinese and alphabetic text. It was found that word skipping rate was insensitive to the interactions of age and frequency and that of age and predictability for Chinese readers, which is a finding consistent with that of previous studies (Wang et al., 2018a; Zhao et al., 2019). However, this finding is inconsistent with that of studies which focused on reading of alphabetic text. Specifically, in English reading, age also promotes the frequency effect on skipping rate of the target word, but does not interact with predictability (Rayner et al., 2006); however, in German reading, it was found that high predictability increased young adult readers’ probability of skipping target words (Kliegl et al., 2004). We also observed no significant interactions between aging, frequency, and predictability for re-fixating rate of target words. This null finding was only partly consistent with the findings of studies on reading of alphabetic text. In English reading, there is no indication that older readers’ re-fixating rate of words might be more likely to be impacted by frequency and predictability. However, in German reading, Kliegl et al. (2004) found that highly predictable words decreased older readers’ probability of multiple fixations on target words. The results of the present study showed that the effects of frequency and predictability on skipping of and re-fixating rate of target words were generally insensitive to age. Thus, it demonstrates a different pattern of the effects of age on skipping and re-fixating across Chinese and alphabetic text reading.

Perhaps the most important contribution of the present study is that it helps understand how text processing affects saccades targeting. We suppose that the reason we could not determine reliable effects of frequency and predictability, and their interaction, on both the length of incoming/outgoing saccades (although reliable effects of frequency and predictability were observed on log-transformed data in terms of length of outgoing saccades, with a shorter outgoing saccade when predictability or frequency was low), is that each Chinese character subtended a larger visual angle than that used in the study by Liu and his colleagues (1.32° vs 1°, Liu, Reichle & Li, 2015; Liu, Reichle & Li, 2016; Liu et al., 2017a; Liu et al., 2017b; Liu et al., 2018), which may eliminate some effects of parafoveal word processing. Nevertheless, for both raw and log-transformed length measures, a reliable interaction effect of age and predictability on incoming saccade length was observed. Specifically, older readers yielded a different predictability effect on incoming saccade length. Previous research found that young Chinese adult readers could use predictability information for promoting the processing of parafoveal characters of the target word (Su, Liu & Cao, 2016). The predictability of words presumably facilitates the preprocessing of these words (Balota, Pollatsek & Rayner, 1985; White, Rayner & Liversedge, 2005a; Schotter et al., 2015), and the interaction effect of age and predictability on incoming saccade length suggests that older readers either experience a decline in the preprocessing of low predictability target words or they make greater use of context to identify words.

Reliable interaction effects of age and frequency on fixation time (i.e., gaze duration, go-past time, and total reading time) indicated that older people were more susceptible to foveal load manipulated by word frequency (see Henderson & Ferreira, 1990; Henderson & Ferreira, 1990; Kennison & Clifton Jr, 1995; Kliegl, Nuthmann & Engbert, 2006; White, Rayner & Liversedge, 2005b; Kliegl, 2007, for word frequency manipulating foveal load). Corresponding to this, reliable interaction effects of age and frequency on outgoing saccades length was observed. We found a negative frequency effect on outgoing saccades length for older readers, which may be a result of their longer fixation on the infrequency of target words. It should be noted that foveal load affects information that can be extracted from the parafoveal; moreover, word frequency modulated outgoing saccades length also reflect the functional role of parafoveal processing in determining saccade targeting (Liu, Reichle & Li, 2015). Thus, the reliable interaction effects of age and frequency on outgoing saccades length also suggest a decline of word processing in determining eye movement during reading. This discrepancy observed in both older and young adult readers when considering the effect of predictability on incoming saccade length and effects of frequency on outgoing saccades length may suggest decline in word processing of older Chinese readers. Future studies must investigate why age impacts the effects of frequency on outgoing saccades length, which is a measure of relatively later word processing, rather than on incoming saccade length. Our findings suggest that word identification in Chinese text depends on readers first identifying characters that might combine to form a word (Ma, Li & Rayner, 2015; thus, frequency effects of two-character words may emerge slowly in Chinese reading.

Taken together, the interaction of age with frequency and predictability affect the amplitude of saccades, which is an important finding as it suggests that age impacts word processing of, possibly both, in parafoveal and foveal vision. This study particularly aimed to assess the impact of age on interactions involving word frequency and predictability in Chinese reading. The absence of interaction effects between word frequency and predictability on eye movements measures in young adult readers indicated that word frequency and predictability impacted a different stage of word processing; this finding is consistent with previous findings of studies on reading alphabetic text (Ashby, Rayner & Clifton, 2005; Gollan et al., 2011; Hand et al., 2010; Slattery, Staub & Rayner, 2012). Conversely, the reliable interaction effect of the three variables on outgoing saccades length produced an interaction of frequency and predictability that was positive for older readers and negative for young adult readers (notably, the reliable effects of a three-way interaction, namely Predictability × Frequency × Group, on both raw and log-transformed outgoing saccades length were observed). Both parafoveal and foveal lexical processing difficulty is used to determine how far one’s eyes would move during reading (Liu, Reichle & Li, 2015; Liu, Reichle & Li, 2016; Liu et al., 2017a; Liu et al., 2017b; Liu et al., 2018). Significant interaction effects of Group × Predictability on incoming saccade length suggests a decline in parafoveal lexical processing, while reliable interaction effects of Group × Frequency on outgoing saccades length suggests a decline in foveal lexical processing for older readers. Therefore, this three-away interaction effect on outgoing saccades length may result from a decline in both parafoveal and foveal lexical processing for old readers.

Several limitations of the present study must be acknowledged. First, the eye-tracker we used has a relatively low spatial and temporal resolution than others. Although this may not be a fatal defect, upon comparison with the methods used in previous studies, we believe that this needs be acknowledged. Second, comprehension scores were lower for older adult readers. It is unlikely that older adult readers did not comprehend sentences because they were all retired teaching staff from the university; they were not very old. Unfamiliarity with computer equipment could have also affected their manual responses. The final limitation is related to validity of the findings. The launch site of impeding saccade may systematically vary across young and older adults, results of saccade length are also likely be systematically affected by higher re-fixation rates of the older adults. But we focused on the interactions of predictability and frequency with age on these two kind of impeding saccade length, the results of reliable interactions must be, at least partly, resulting from these systematical differences. We tested our hypothesis multiple times, and came to the conclusion that using Bonferroni corrections was the most effective method to validate our results (Von der Malsburg & Angele, 2016). Considering our hypothesis that age interacts with linguistic characteristics based on saccade length in Chinese reading, we observed three interactions for each of the two saccade length measures; therefore an alpha of 0.0083 is appropriate in this context (new *α* =0.05∕6 ≈ 0.0083). Barring the three-away interaction effects on log-transformed outgoing saccades length, and Group × Predictability interaction effect on raw data and incoming saccade length, we have interpreted various null results of interactions in our discussion. Although the extent to which extent statistic power has been reduced by Bonferroni corrections is unclear, a reliable three-way interaction may be owing to the two interactions of age with frequency and with predictability. Discussing these “null” results have has helped clarify the interactions effects of age on impeding saccade length.

The present study, and other similar studies, may contribute to a better understanding of language specific characters and how reading behaviors change with age, specifically in readers of the Chinese text. In summary, we found that effects related to age, word frequency, and predictability appear to be similar across alphabetic and non-alphabetic text, when assessed with measures of fixation time. Measures of saccade length maybe the most informative for understanding how age impacts word processing in Chinese readers. Specifically, a unique pattern of results was observed in terms of measures of incoming and outgoing saccades length. Thus, present study provides evidence suggesting that these age-related effects differ qualitatively across alphabetic text reading. Previous research has already elaborated on this qualitative difference. Paterson et al. (2015) found that young and older adult readers of alphabetic text demonstrated similar patterns of landing position on both short and long words. This finding suggests that eye guidance is preserved in older adult readers during alphabetic text reading. We suppose that this was due to differences in the behaviors of alphabetic text and Chinese text readers. Specifically, reading alphabetic text requires the use of visual cues for guidance on where to move one’s eyes, and requires the reader to focus on a target word regardless of word length (Rayner, 1998; Rayner, 2009). Whereas, reading Chinese text requires eye movements, that is saccade targeting, to be modulated by the parafoveal and foveal lexical processing (Liu, Reichle & Li, 2015; Liu, Reichle & Li, 2016; Liu et al., 2017a; Liu et al., 2017b). Our findings demonstrate that age interacts with lexical processing to effect saccade length. Ultimately, observing effects of age on eye movements while reading in Chinese may help in further understanding of the characteristics that underlie changes in reading behaviors over time.

## Conclusions

Results of present study suggest that the word processing function of older Chinese readers in terms of saccade targeting declines with age.

##  Supplemental Information

10.7717/peerj.8860/supp-1 Supplemental Information 1 The raw data exported from the eye movement tracker applied for data analyses and preparation for Tables 4 and 5

10.7717/peerj.8860/supp-2 Supplemental Information 2 Variables in the database for the raw data for Tables 4 and 5

10.7717/peerj.8860/supp-3 Supplemental Information 3 The raw data of first fixation duration, gaze duration, total reading time, Go-past time probability of skipping and re-fixating on a target word and incoming saccade length Exported from the eye movement tracker applied for data analyses and preparation for Tables 6, 7 and 8.

10.7717/peerj.8860/supp-4 Supplemental Information 4 Variables in the database for the raw data for Tables 6, 7 and 8

10.7717/peerj.8860/supp-5 Supplemental Information 5 The raw data of outgoing saccades length (OSL) exported from the eye movement tracker applied for data analyses and preparation for Tables 6 and 8

10.7717/peerj.8860/supp-6 Supplemental Information 6 Variables in the database for the raw data for Tables 6 and 8

10.7717/peerj.8860/supp-7 Supplemental Information 7 Comprehension accuracy

10.7717/peerj.8860/supp-8 Supplemental Information 8 R analysis Code

## Additional Information and Declarations

### Data Availability

The following information was supplied regarding data availability: Raw data are available as Supplementary Files.
