# Supplementary material for: Interaction effects of aging, word frequency, and predictability on saccade length in Chinese reading
Source: PeerJ. 2020 Apr 1;8:e8860. doi: 10.7717/peerj.8860 (PMC7127474; doi:10.7717/peerj.8860)
Supplement: Supplemental Information 2 [file peerj-08-8860-s002.doc]

Readme file of “CSVPreFreAging120TrailReport”

Columns used for data analysis were: group, id, freq, item, pred,

Amplitude1

group: 1= young adults, 2=older adults;

id= identification number of subjects;

freq=frequency of target words, H= high, L=low;

item= identification number of frame sentences;

pred=predictability of target words, H=predictable, L= unpredictable;

afd=average fixation duration (AFD; mean duration of all fixation while reading a sentence)

asa=average saccade length (ASA; mean length of all saccades)

Srts=entence reading time (SRT),

fc=fixation count (FC),

regno=regression number (RegNO; backward saccades number),
