# Supplementary material for: Interaction effects of aging, word frequency, and predictability on saccade length in Chinese reading
Source: PeerJ. 2020 Apr 1;8:e8860. doi: 10.7717/peerj.8860 (PMC7127474; doi:10.7717/peerj.8860)
Supplement: Supplemental Information 4 [file peerj-08-8860-s004.doc]

Readme file of “CSVPreFreAging120IASReportTTsub.csv”

Columns used for data analysis were: group, id, freq, item, pred,

ref ,skip, idt, ffd, gd, amplitude, go-past,

group: 1= young adults, 2=older adults;

id= identification number of subjects;

freq=frequency of target words, H= high, L=low;

item= identification number of frame sentences;

pred=predictability of target words, H=predictable, L= unpredictable;

ref= whether or not target was re-fixated, 0=no,1=yes

skip= whether or not target was skipped, 0=no,1=yes

idt= sum of all fixations’ duration

ffd= the duration of the first fixation on the word irrespective of the number of fixations

gd= the sum of all fixations’ duration on the word before moving to another word

Amplitude=incoming saccade length (ISL; length of the first-pass progressive saccade resulting in fixation on the target word)

go-past = sum of fixation duration from when the current area of interest is first fixated upon until one’s eyes enter an interest area with a higher

Logidt= log-transformed of total reading time

Logffd= log-transformed of first fixation duration

Loggd= log-transformed of gaze duration
