# Supplementary material for: Interaction effects of aging, word frequency, and predictability on saccade length in Chinese reading
Source: PeerJ. 2020 Apr 1;8:e8860. doi: 10.7717/peerj.8860 (PMC7127474; doi:10.7717/peerj.8860)
Supplement: Supplemental Information 6 [file peerj-08-8860-s006.doc]

Readme file of “CSVPreFreAging120IASSacT-PEERJ.csv”

Columns used for data analysis were: group, id, freq, item, pred,

Amplitude1

group: 1= young adults, 2=older adults;

id= identification number of subjects;

freq=frequency of target words, H= high, L=low;

item= identification number of frame sentences;

pred=predictability of target words, H=predictable, L= unpredictable;

amplitude1= outgoing saccades length (OSL; length of the first-pass progressive saccade launched away from the target word).
