# Supplementary material for: Interaction effects of aging, word frequency, and predictability on saccade length in Chinese reading
Source: PeerJ. 2020 Apr 1;8:e8860. doi: 10.7717/peerj.8860 (PMC7127474; doi:10.7717/peerj.8860)
Supplement: Supplemental Information 8 [file peerj-08-8860-s008.doc]

library(languageR)

library(lme4)

library(lattice)

library(MASS)

library(plyr)

library(lmerTest)

datafile = read.csv(file.choose(), sep = ",", dec = ".")

str(datafile)

datafile$group=as.factor(datafile$group)

datafile$logffd <- log(with(datafile,ffd))

datafile$loggd <- log(with(datafile,gd))

datafile$logidt <- log(with(datafile,idt))

datafile$loggopast <- log(with(datafile,gopast))

datafile$logamplitude <- log(with(datafile,amplitude))

contrasts(datafile$group) <- contr.sdif(2)

contrasts(datafile$pred) <- contr.sdif(2)

contrasts(datafile$freq) <- contr.sdif(2)

ffd.lmer1= lmer(ffd ~ pred * freq * group+ (1|item) + (1|id), datafile)

summary(ffd.lmer1, corr = FALSE)

logffd.lmer1= lmer(logffd ~ pred * freq * group+ (1|item) + (1|id), datafile)

summary(logffd.lmer1, corr = FALSE)

gd.lmer1= lmer(gd ~ pred * freq * group+ (1|item) + (1|id), datafile)

summary(gd.lmer1, corr = FALSE)

gd.lmer2= lmer(gd ~ pred * freq * group + (1|id), datafile)

summary(gd.lmer2, corr = FALSE)

loggd.lmer1= lmer(loggd ~ pred * freq * group+ (1|item) + (1|id), datafile)

summary(loggd.lmer1, corr = FALSE)

idt.lmer1= lmer(idt ~ pred * freq * group+ (1|item) + (1|id), datafile)

summary(idt.lmer1, corr = FALSE)

logidt.lmer1= lmer(logidt ~ pred * freq * group+ (1|item) + (1|id), datafile)

summary(logidt.lmer1, corr = FALSE)

logidt.lmer2= lmer(logidt ~ pred * freq * group + (1|id), datafile)

summary(logidt.lmer2, corr = FALSE)

gopast.lmer1= lmer(gopast ~ pred * freq * group+ (1|item) + (1|id), datafile)

summary(gopast.lmer1, corr = FALSE)

loggopast.lmer1= lmer(loggopast ~ pred * freq * group+ (1|item) + (1|id), datafile)

summary(loggopast.lmer1, corr = FALSE)

amplitude.lmer1= lmer(amplitude ~ pred * freq * group+ (1|item) + (1|id), datafile)

summary(amplitude.lmer1, corr = FALSE)

logamplitude.lmer1= lmer(logamplitude ~ pred * freq * group+ (1|item) + (1|id), datafile)

summary(logamplitude.lmer1, corr = FALSE)

skip.glmer1= glmer(skip ~ pred * freq * group +(1|item) + (1|id), datafile, family = binomial)

summary(skip.glmer1, corr = FALSE)

ref.glmer1= glmer(ref ~ pred * freq * group +(1|item) + (1|id), datafile, family = binomial)

summary(ref.glmer1, corr = FALSE)

reg.glmer1= glmer(reg ~ pred * freq * group +(1|item) + (1|id), datafile, family = binomial)

summary(reg.glmer1, corr = FALSE)
